# Thyroid hormone receptor beta is critical for intestinal remodeling during *Xenopus tropicalis* metamorphosis

**DOI:** 10.1186/s13578-020-00411-5

**Published:** 2020-03-27

**Authors:** Yuki Shibata, Yuta Tanizaki, Yun-Bo Shi

**Affiliations:** Section on Molecular Morphogenesis, National Institute of Child Health and Human Development, National Institutes of Health, Bethesda, MD 20892 USA

**Keywords:** Thyroid hormone receptor, *Xenopus tropicalis*, Anuran metamorphosis, Intestinal remodeling, Stem cells, Apoptosis

## Abstract

**Background:**

Thyroid hormone (T3) is critical for development in all vertebrates. The mechanism underlying T3 effect has been difficult to study due to the uterus-enclosed nature of mammalian embryos. Anuran metamorphosis, which is dependent on T3 but independent of maternal influence, is an excellent model to study the roles of T3 and its receptors (TRs) during vertebrate development. We and others have reported various effects of *TR* knockout (*TRα* and *TRβ*) during *Xenopus tropicalis* development. However, these studies were largely focused on external morphology.

**Results:**

We have generated *TRβ* knockout animals containing an out-frame-mutation of 5 base deletion by using the CRISPR/Cas9 system and observed that *TRβ* knockout does not affect premetamorphic tadpole development. We have found that the basal expression of direct T3-inducible genes is increased but their upregulation by T3 is reduced in the intestine of premetamorphic homozygous *TRβ* knockout animals, accompanied by reduced target binding by TR. More importantly, we have observed reduced adult stem cell proliferation and larval epithelial apoptosis in the intestine during T3-induced metamorphosis.

**Conclusions:**

Our data suggest that *TRβ* plays a critical role in intestinal remodeling during metamorphosis.

## Introduction

Thyroid hormone (T3) is essential for normal development in all vertebrates, particularly the postembryonic developmental period around birth in mammals and metamorphosis in amphibians when plasma T3 levels peak [1–9]. T3 deficiency in human due to iodine deficiency causes congenital hypothyroidism with severe development problems including impaired mental function, retarded physical development and goiter formation [3, 6]. The developmental effects of T3 are believed to be mediated mainly, if not exclusively, by T3 receptors (TRs). When T3 is absent, the unliganded TRs, which can form heterodimers with 9-cis-retinoic acid receptors (RXRs), bind to T3-response elements (TREs) and recruit corepressors to repress T3-inducible genes. After T3 binding to TRs, the liganded TRs recruit coactivators to induce the expression of these T3 target genes [1, 2, 4, 10–23]. A number of mouse models with individual *TRα*, *TRβ*, or *TRα/TRβ* double knockout have been produced and revealed different TR-isoform-dependent, tissue-specific defects in the adult, supporting important roles of the receptors in regulating T3 signaling during mammalian development [24–31].

We have been studying amphibian metamorphosis as a model system for understanding the regulation of postembryonic vertebrate adult organ development by T3. During metamorphosis in anurans such as the pseudo-tetraploid *Xenopus laevis* and its highly related diploid species *Xenopus tropicalis*, T3 induces dramatic tissue modifications in essentially all tissues/organs [3, 4, 32–36]. Of particular interest among them is intestinal remodeling. In adult vertebrates, the intestinal epithelium undergoes constant self-renewal through stem cell proliferation in the crypt (mammals) or bottom of the epithelial fold (anurans) and the eventual apoptotic death of the differentiated epithelial cells, mainly at the tip of the villus (mammals) or fold (anurans) [37–44]. Interestingly, this self-renewing system is established during intestinal metamorphosis in anurans or the postembryonic period in mammals [45, 46]. In anurans such as *Xenopus tropicalis*, the tadpole intestine is a simple tubular structure with the epithelium surrounded by thin layers of connective tissue and muscles [38, 47]. During metamorphosis, there is a near total degeneration of the larval epithelium, accompanied by concurrent de novo development of the adult epithelial stem cells, which subsequently proliferate and differentiate to from a complex adult epithelial structure resembling that in adult mammals [38, 39, 45, 46, 48–51]. The total dependence of this process on T3 provides a unique opportunity to analyze the roles of unliganded vs. liganded TR during adult organ development, particularly stem cell formation.

Interestingly, when analyzed in the intestine or whole tadpoles, *TRα* is expressed at high levels from premetamorphosis to the end of metamorphosis while *TRβ* expression is low during premetamorphosis but is induced as a direct T3 target gene to peak at the climax of metamorphosis [36, 52–55]. Consistently, gene knockout studies in *Xenopus tropicalis* have shown that *TRα* single knockout accelerates premetamorphic tadpole growth and development (based on hindlimb morphology), likely due to the removal of unliganded TRα which leads to depression of T3 target genes [56, 57]. In addition, there is a delay in intestinal remodeling relative to the external morphological changes in the *TRα* single knockout tadpoles [56, 57]. On the other hands, *TRβ* single knockout tadpoles are normal in premetamorphic development based on external morphology but have delayed tail regression during metamorphosis [58, 59]. On the other hand, the hindlimb development and intestinal remodeling have only relatively subtle differences between the *TRβ* knockout and wild type animals during natural metamorphosis [58, 59], a very surprising finding, especially for the intestine given the strong upregulation of *TRβ* during intestinal metamorphosis. Finally, our recent *TR* double knockout study have revealed that removing all TRs leads to precociously adult organ development but prevents/inhibits larval tissue degeneration during metamorphosis with the tadpoles stalling their development at climax of metamorphosis for up to two weeks before death prior to the completion of tail resorption [60]. These findings suggested that TRs play critical, isotype-specific roles during metamorphosis. On the other hand, given the different effects of *TR* knockouts on different organs, it is difficult to interpret whether the delay in intestinal remodeling during natural metamorphosis is due to direct effect on the intestine or indirect consequence of the different effects of the knockouts on different organs, e.g., accelerated metamorphosis of the external organs, which is used to judge developmental stages, may make intestinal remodeling appear delayed relative to external organs even if the knockouts do not affect the intestine directly.

In this study, we have knocked out *Xenopus tropicalis* TRβ gene by using CRISPR/Cas9 genome editing technology and studied age- and stage-matched wild type and knockout animals during T3-induced metamorphosis to avoid the potential artifact due to comparing animals of different ages as during natural metamorphosis. We observed no development defects due to the knockout from embryogenesis to premetamorphosis even though there was derepression of T3 target genes in the *TRβ*^(*−/−*)^ premetamorphic tadpoles. Examination of the tadpoles at stage 54 with or without T3 has revealed that *TRβ*^(*−/−*)^ tadpoles have reduced responses to exogenous T3 treatment, particularly with regard to T3-induced gill resorption and reshaping of the head to a pointy structure. Importantly, T3-induced intestinal remodeling, including length reduction, adult stem cell development and proliferation, and larval epithelial cell death are all inhibited by the *TRβ* knockout, accompanied by reduction in both target binding by TR and T3-upregulation of target genes in the intestine. Our data suggest that TRβ is upregulated by T3 in the intestine and in turn feeds back positively to activate downstream genes in the intestine to promote larval cells apoptosis and adult epithelial stem cell development and proliferation.

## Results and discussion

### Generation of *TRβ* knockout animals

**Fig. 1:**
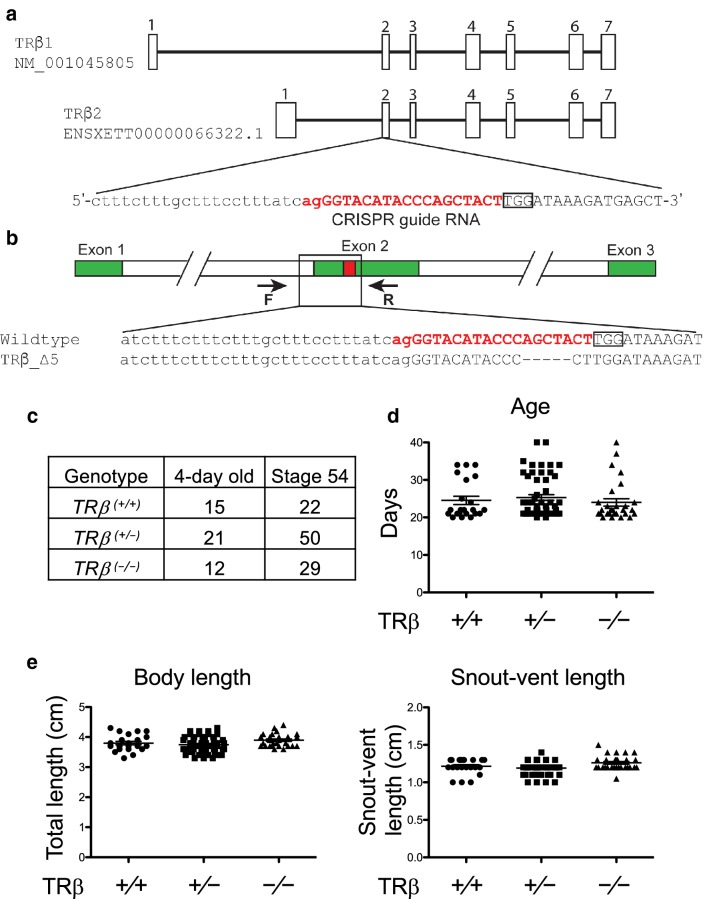
Knocking out *TRβ* gene in *Xenopus tropicalis* does not affect early development. **a** Genomic structure of *X. tropicalis TRβ* gene and the CRISPR-short guide RNA (sgRNA) targeting *TRβ*. There are two known transcripts for *X. tropicalis TRβ*, each with 7 exons (boxes). The *TRβ* specific sgRNA was designed to target exon 2 present in both transcripts. The sgRNA sequences are shown in red. **b** Schematic diagram depicting the sequence of the sgRNA targeted region in the wild type and a *TRβ* mutant (5 base out-of-frame deletion) animal. Arrows represent primers used for genotyping: the forward primer F and the reverse primer R, respectively. **c** Mendelian distribution of 4-day old (around stage 45/46) or stage 54 tadpoles obtained from mating two *TRβ*^(+/−)^ animals. Genotyping PCR was carried out by using tail tip genomic DNA of randomly selected 4-day old (stage 45/46) and stage 54 tadpoles, the onset of metamorphosis, and the results were close to the expected Mendelian distribution for the three expected genotypes. **d** Knocking out *TRβ* does not affect the developmental rate up to the onset of metamorphosis (stage 54). The time in days for each animal obtained from mating two *TRβ*^(+/−)^ frogs to reach stage 54 was recorded and presented with the mean, marked as a line, and standard error (SE). No significant difference was observed for the 3 genotypes. **e** Knocking out TRβ does not affect the animal size and morphology at stage 54. The total body length and snout-vent length were measured on randomly selected animals at stage 54 and presented with the mean, marked as a line, and standard error (SE). No significant difference was observed for the 3 genotypes

We previously reported the adaptation of Clustered Regularly Interspaced Short Palindromic Repeats (CRISPR) genome editing technology to knockout *Xenopus tropicalis TRβ* in the *TRα* knockout heterozygous animals to generate *TR* double knockout tadpoles [60]. We also used this approach to generate *TRβ* single knockout animals. Briefly, we co-injected *Cas9* mRNA and CRISPR-short guide RNA (sgRNA) targeting exon 2 that encodes part of the DNA binding domain of TRβ into one-cell stage of fertilized eggs obtained from a *TRα* heterozygous frog [*TRα*^(+/−)^*TRβ*^(+/+)^] and wild type frog [TRα^(+/+)^TRβ^(+/+)^] (Fig. 1a). We selected offspring (F0 generation) with wild type *TRα* as genotyped by PCR [61] and containing mosaic mutations in *TRβ* [*TRα*^(+*/*+*)*^*TRβ*^*mosaic*^] as determined by sequence analysis [60]. After sexual maturation of F0 animals, we crossed them with wild type animals and obtained *TRβ* heterozygous knockout animals (F1 generation) [*TRβ*^(+/−)^] with a 5 base out-of-frame mutation (Fig. 1b). We raised some of F1 *TRβ* heterozygous mutant animals to sexual maturity and generated F2 generation, *TRβ* homozygous knockout animals [*TRβ*^(−/−)^] by intercrossing F1 heterozygous mutant frogs.

### *TRβ* knockout does not affect embryogenesis and premetamorphosis despite some derepression of T3 target genes expression in premetamorphic tadpoles

To analyze the effects of *TRβ* knockout on *Xenopus* development, we genotyped F2 siblings from mating two *TRβ* heterozygous animals, 48 tadpoles at 4 days old (around stages 45/46, the onset of feeding) and 101 tadpoles at developmental stage 54, respectively. Genotyping data revealed a Mendelian distribution of the three expected genotypes at both time points (Fig. 1c). We next recorded their age when individual tadpoles reached stage 54 according to their external morphology and measured the total body length and snout-vent length (Fig. 1d, e). There was no significant difference in the three parameters among the three genotypes and all animals had normal morphology. These results suggest that *TRβ* knockout had no effect on embryogenesis and premetamorphosis, consistent with the very low levels of *TRβ* expression during these developmental periods [52, 54, 55].

### T3 direct target genes are derepressed in the intestine of *TRβ* knockout tadpoles at stage 54, accompanied by reduced TR binding

**Fig. 2:**
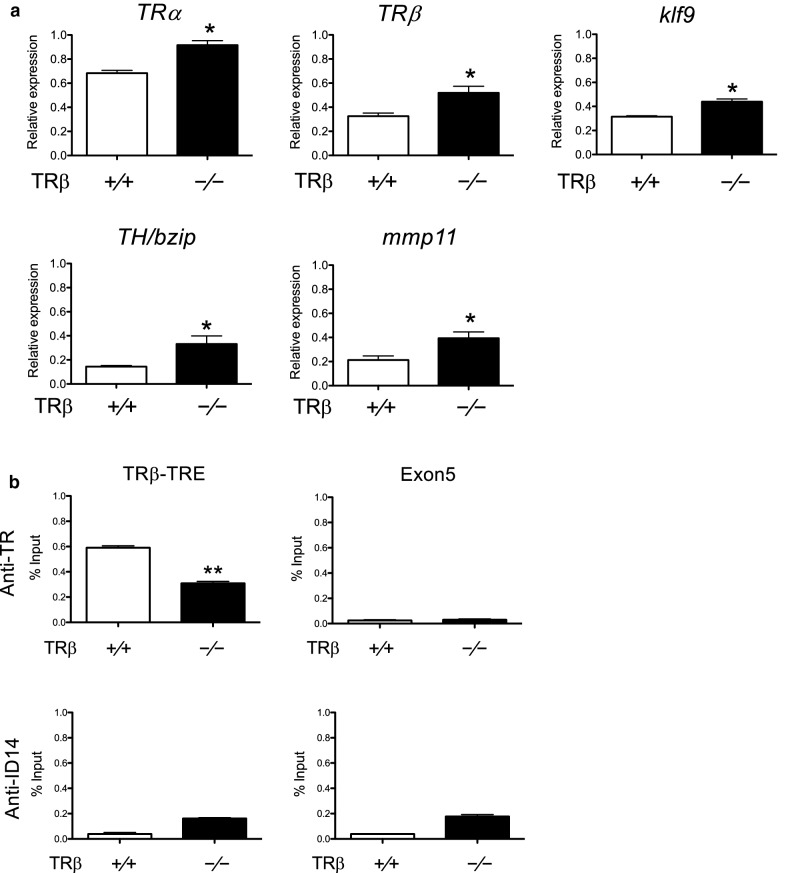
Basal expression of *TRα* and known T3 target genes in premetamorphic tadpoles is increased in *TRβ* knockout intestine, accompanied by reduced TR binding to the endogenous TRE region. **a***TRβ* knockout increased *TRα* and T3 target gene expression in premetamorphic tadpoles. Total RNA of three different tissues, intestine, tail and hindlimb, of stage 54 tadpoles of the three genotypes, was used for qRT-PCR analysis of the expression of *TRα* and four well-known T3 direct target genes: *TRβ*, *klf9*, *mmp11* and *TH/bzip*. The expression levels were normalized against that of *rpl8*. Asterisks denote statistically significant differences (*P* < 0.05). Note that in the knockout tadpoles, *TRβ* mRNA, which had an out-of-frame mutation, was expressed at a higher level, likely due to partial de-repression caused by the lack of TRβ protein expression, just like the other target genes*.***b** ChIP assay reveals reduced TR binding at the TRE region. The intestine sample obtained from at least five tadpoles of both genotypes, wild type and *TRβ* knockout, at stage 54 and were homogenized together for ChIP assay with antibodies against TR and ID14 as a negative control. The presence of the TRE region or the exon 5, as a negative control, of *TRβ* gene was determined by PCR. Asterisks (**) indicates statistically significant differences (*P* < 0.01)

To investigate the effects of the knockout on the animal intestine, we analyzed the expression of several well-known direct T3 target genes, *TRβ* [53], *klf9* [62], *mmp11 *[63], and *TH/bzip* [64], in the intestine of premetamorphic wild type and *TRβ* knockout tadpoles. The results showed that the expression of all these genes were increased significantly in *TRβ*^(−/−)^ tadpoles (Fig. 2a) (note that the knockout animals expressed a non-functional mutant *TRα* mRNA). To see if there were any changes in *TRα* expression, we analyzed *TRα* mRNA level and found a small increase in the intestine of premetamorphic *TRβ* knockout tadpoles compared to the wild type ones (Fig. 2a). To determine whether the increase in T3 target gene expression was due to partial derepression due to the loss of TRβ in the mutant animals, we analyzed TR binding to the well-characterized TRE in the *TRβ* gene in the intestine of stage 54 tadpoles by ChIP assay [56, 61]. As shown in Fig. 2b, TR binding to the TRE region was significantly decreased in *TRβ* knockout animals compared with wild type animals. These results suggest that the reduction of TR binding, with the remaining TR binding due to *TRα*, likely causes a reduction in the repression by unliganded TR, thus leading to the derepression of T3 target genes in premetamorphic tadpoles. Since *TRα* mRNA level also rises during intestinal metamorphosis, the increase in *TRα* mRNA level in the premetamorphic *TRβ* knockout tadpoles may also be due to this derepression, although it is not yet known if *TRα* is a directly T3 target gene.

### Premetamorphic *TRβ* knockout tadpoles have a poor intestinal response to exogenous T3 treatment

**Fig. 3:**
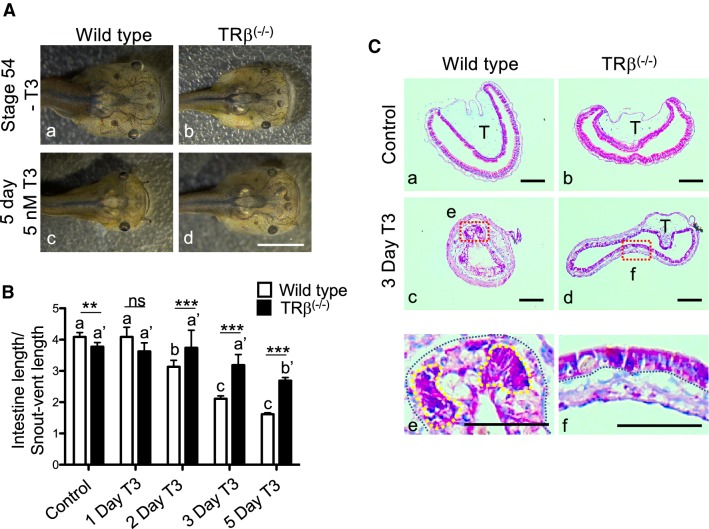
Homozygous *TRβ* mutant tadpoles have drastically reduced responses to exogenous T3. **A** Representative photos of tadpoles treated with 5 nM T3 for 5 days. Age-matched stage 54 tadpoles were randomly selected and kept in a 4L plastic container with (c and d) or without (a and b) 5 nM T3 treatment for 5 days. They were then genotyped and photographed dorsally. Note that after T3 treatment, the wild type tadpole (c) showed obviously metamorphic changes and was morphologically distinct from *TRβ*^(−/−)^ tadpoles (d), which was similar to the untreated tadpoles. Bars: 1 mm. **B***TRβ* knockout tadpoles had reduced intestinal shortening in response to T3. The lengths of the intestine of the tadpoles as treated in **A** were measured and normalized against the snout-vent length. Note that after T3 treatment, the intestine was significantly shortened in wild type tadpoles but this change was reduced and delayed in the *TRβ*^(−/−)^ tadpoles. Different lower-case letters denote statistically significant differences (*P* < 0.05) when compared in each genotypes and asterisks (** or ***) indicate a significant difference when compared to the wild type group at the indicated days for T3 treatment (*P* < 0.01 or *P* < 0.001). ns: no significant difference. **C** T3 treatment failed to induce epithelial remodeling in the *TRβ* knockout tadpoles. Age-matched tadpoles at stage 54 were treated with 5 nM T3 for 3 days. Cross-sections of the intestine isolated from wild type (a, c and e) and *TRβ* knockout (b, d and f) tadpoles were stained with MGPY (methyl green-pyronin Y). Dashed red boxes (c and d) indicate the higher magnification (e and f), respectively. The black-dotted lines depict the epithelium-mesenchyme boundary, drawn based on morphological differences between epithelial cells and mesenchyme cells in the pictures of the stained tissues. The yellow-dotted lines indicate the cluster of proliferating epithelial cell which are well stained in wild type tadpoles after 3 days T3 treatment (e). *T* typhlosole. Bars: 100 µm

To investigate whether *TRβ* knockout affects intestinal response to exogenous T3 treatment, wild type and *TRβ* knockout tadpoles at stage 54 were treated with or without T3 for up to 5 days, a long term T3 treatment that can induce the external morphological change such as the formation of a pointy head, the resorption of the gills, and intestinal length reduction in wild type animals (Fig. 3A). However, *TRβ* knockout tadpoles showed little change after T3 treatment (Fig. 3A). In addition, there was also a delayed reduction in the intestinal length (Fig. 3B). To examine the cellular changes, we stained the intestinal cross-sections with methyl green pyronin Y (MGPY), a mixture of methyl green, which binds to DNA, and pyronin Y, which binds to RNA and thus labels very strongly the proliferating cells but poorly the apoptotic cells [51]. As expected, the intestine at stages 54 of both genotypes were composed of mainly a monolayer of larval epithelial cells surrounded by thin connective tissue and muscles, with only a single epithelial fold, the typhlosole (Fig. 3C a, b). After 3 days T3 treatment, as the larval epithelial cells underwent apoptosis, they became poorly labeled, while clusters of strongly labeled proliferating adult stem cells were found between the connective tissue and dying larval epithelial cells in wild type tadpoles (yellow surrounded Fig. 3C e). The intestine of the *TRβ* knockout tadpoles after 3 days T3 treatment resembled that in the wild type or *TRβ* knockout stage 54 tadpoles without T3 treatment (Fig. 3C d, f), indicating a lack of or drastically reduced response of the mutant intestine to exogenous T3. These results suggest that TRβ is required for the intestinal remodeling in response to T3.

### Decreased induction of T3 response gene expression is associated with reduced TR binding in the intestine of *TRβ* knockout tadpoles after short term T3 treatment

**Fig. 4:**
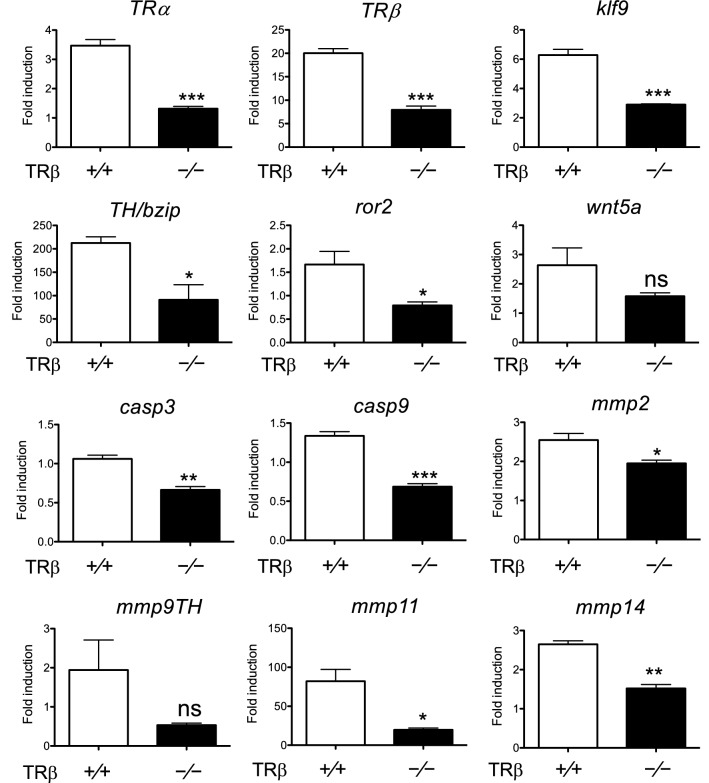
T3 response genes fail to be upregulated in the intestine of *TRβ* knockout tadpoles during metamorphosis. Total RNA was isolated from the intestine of wild type and *TRβ* knockout tadpoles at stage 54 treated with or without 10 nM T3 for 18 h and used for real-time RT-PCR analysis of the expression of *TRα* and known T3 direct target genes: *TRβ*, *klf9*, and *TH/bzip*; genes related to ECM remodeling: *mmp2*, *mmp9*, *mmp11*, and *mmp14*; apoptosis-related genes: caspase 3 (*casp3*) and caspase 9 (*casp9*); and cell dedifferentiation-related genes: *ror2* and *wnt5a*. The mRNA levels were normalized against that of *rpl8*. The groups included 5 wild type and 5 *TRβ* homozygous [*TRβ*^*(−/−)*^] animals. Note that T3 upregulated the expression of nearly all genes in the wild type animals but this upregulation was drastically reduced or abolished in the knockout animals. Asterisks (*, ** and ***) indicate a significant difference vs wild type as determined by *s*tudent*-t* test (*P* < 0.05, *P* < 0.01 and *P* < 0.001). *ns* no significant difference

**Fig. 5:**
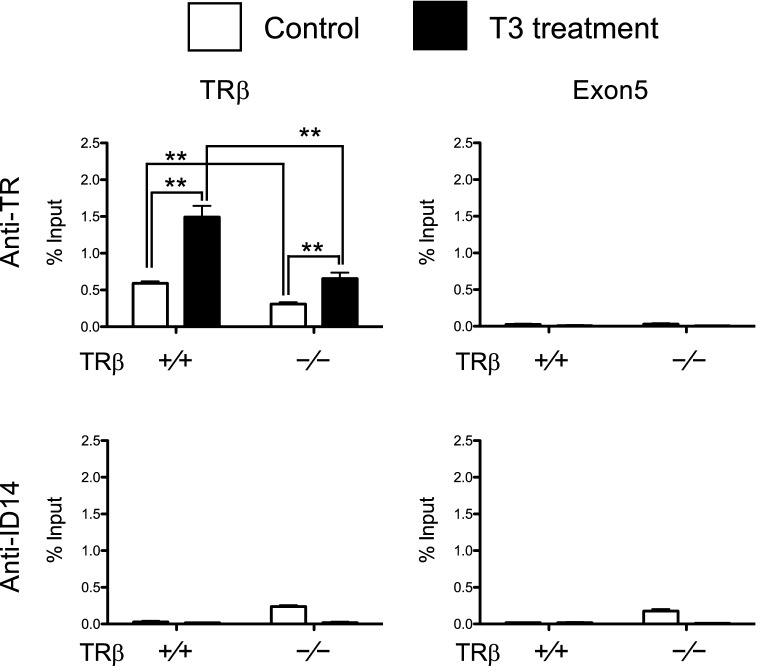
*TRβ* knockout reduces TR binding to the TRE region in the tadpole intestine after T3 treatment. Five or more age-matched wild type and *TRβ* knockout tadpoles at stage 54 were kept in 4-l plastic container and treated with or without 10 nM T3 for 18 h. The chromatin of the intestine was isolated and immunoprecipitated with antibodies against TR and ID14 as a negative control. The immunoprecipitated DNA was analyzed by real-time PCR for the presence of the TRE region or the exon 5, which lacks any TRE, of the *TRβ* gene. Note that TR binding to the TRE region was significantly reduced in the intestine of *TRβ* knockout tadpoles. Two asterisks (**) indicate statistically significant differences (*P* < 0.01)

To determine if the lack of intestinal remodeling in the knockout animals was due to reduced target gene expression, we analyzed the expression of some known T3 response genes during intestinal metamorphosis. As shown in Fig. 4, known T3 upregulated genes, including transcription factors *TRα*, *TRβ*, *klf9* and *TH/bzip*; and matrix metalloproteinase (MMP) genes, *mmp2*, *mmp9th*, *mmp11* and *mmp14*, all but *mmp9th* had significantly reduced induction by T3 in the intestine of *TRβ* knockout tadpoles at stage 54 compared to the wild type intestine. Analyses of two apoptosis-related genes, caspase 3 (*casp3*) and caspase 9 (*casp9*) [65], and dedifferentiation related genes, *ror2* and *wnt5a* [66], found that all but *wnt5a* had significantly lower expression in the knockout intestine compared to the wild type intestine (Fig. 4). In addition, ChIP assays on the T3-treated animal intestine showed that *TRβ* knockout tadpole intestine had significantly reduced TR binding to the TRE in *TRβ* gene, a well-characterized direct T3 target gene in the intestine (Fig. 5). These results suggest that gene activation by liganded TRβ is important for T3-induced intestinal metamorphosis.

### T3-induced epithelial stem cell formation/proliferation and larval epithelial cell death is inhibited/reduced in homozygous *TRβ* knockout tadpole intestine

**Fig. 6:**
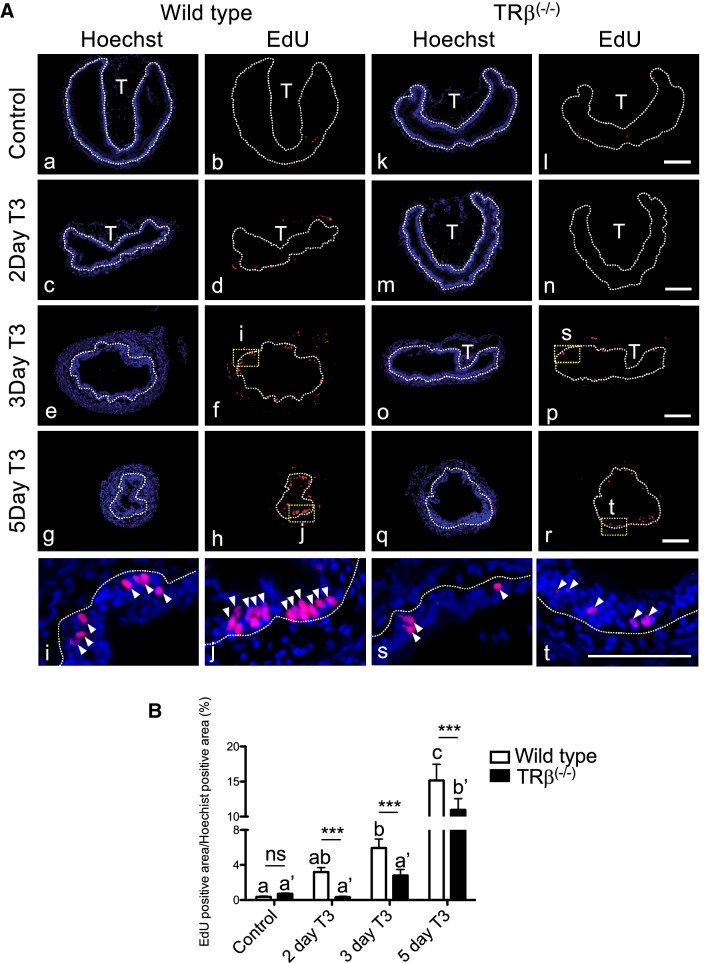
Knocking out of *TRβ* tadpoles reduces intestinal stem cell proliferation during T3-induced metamorphosis. **A** Reduced EdU labeling in TRβ knockout tadpoles after T3 treatment. Wild type (a–j) and *TRβ* knockout (k–t) tadpoles at stage 54 were treated with or without 5 nM T3 treatment for up to 5 days. The animals were injected with EdU solution for 30 min before being sacrificed. Cross-sections of the intestine were prepared and stained with Hoechst 33342 for DNA and EdU for proliferating cells. The dotted lines depict the epithelium-mesenchyme boundary, drawn based on morphological differences between epithelial cells and mesenchyme cells in the pictures of the stained tissues, under enhanced contrast and/or brightness by using Photoshop, if needed. Dashed yellow boxes (f, h, p and r) indicate the higher magnification (i, j, s and t), respectively. EdU: red, and Hoechst: blue. T: typhlosole. At least 3 tadpoles were analyzed for each genotype. Bars: 100 µm. **B** EdU positive area was reduced in the intestine of *TRβ* homozygous knockout animals. The EdU positive area in **A** was quantified and normalized against the Hoechst 33342 positive area. Different lower case letters denote statistically significant differences (*P* < 0.05) when compared in a single genotype and asterisks (***) indicate a significant difference between the two genotypes (*P* < 0.001). *ns* no significant difference

In addition to the reduction in the length of the intestine, the major changes during intestinal metamorphosis involves the near complete degeneration of the larval epithelial cells and concurrent development of adult epithelial stem cells, following by their proliferation and differentiation to form the adult epithelium [38, 39, 45, 46, 48–51]. Thus, we next investigated cell proliferation by in vivo labeling with 5-ethynyl-2′-deoxyuridine (EdU). Wild type and *TRβ* knockout tadpoles at stage 54 were treated with T3 for up to 5 days to induce metamorphosis and then injected with 1.25 µl of EdU solution 10 mg/ml into the body of each tadpole [67]. The animals were subsequently sacrificed and sectioned for EdU staining to detect EdU positive cells, and for TUNEL staining to detect apoptotic cells in the intestine. EdU staining clearly detected active cell proliferation in the intestine in both wild type and knockout tadpole intestine (Fig. 6A). In the wild type intestine, the number of EdU positive cells gradually increased in response to T3 treatment and clusters of strongly labeled proliferating stem cells appeared after 3 days T3 treatment (Figs. 6A i, j). However, in *TRβ* knockout tadpoles, relatively few EdU positive cells were detected with no observable proliferating cell clusters after three days (Fig. 6 s, t). Quantitative measurement of the EdU positive area, normalized against Hoechst positive area, showed that the induction of cell proliferation was reduced in *TRβ* knockout intestine at all time points between 2–5 days of T3 treatment (Fig. 6B).

**Fig. 7:**
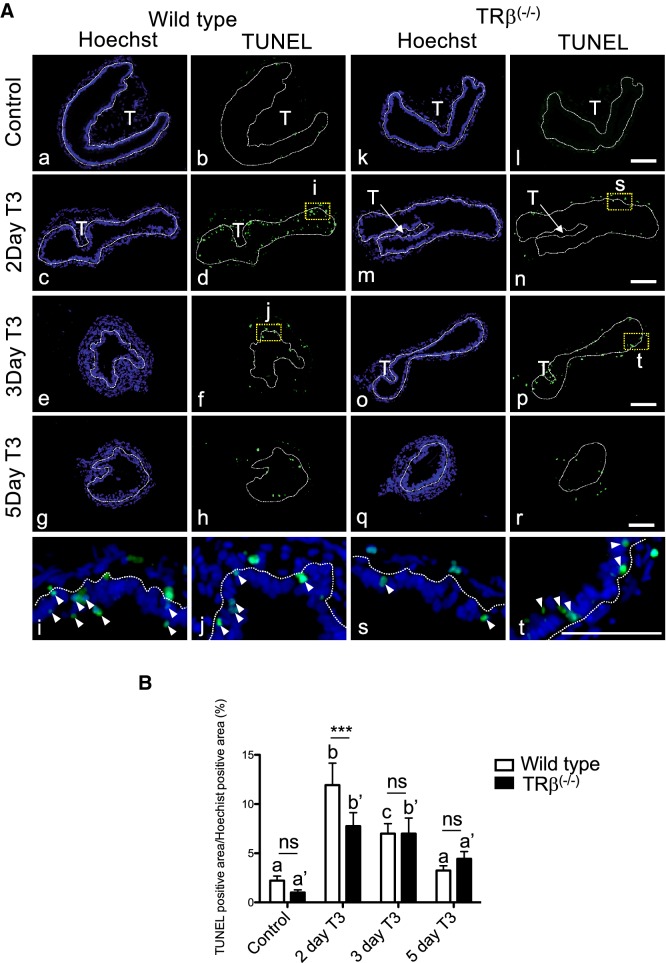
Knocking out of *TRβ* tadpoles reduces epithelial cell death after T3 treatment. **A** Reduced TUNEL labeling for apoptotic cells in TRβ knockout tadpoles. Cross-sections of the intestine isolated from wild type (a–j) and *TRβ* knockout (k–t) tadpoles at stage 54 treated with or without 5 nM T3 treatment for up to 5 days were subjected to TUNEL labeling for apoptotic cells and Hoechst 33342 staining for DNA. The dotted lines depict the epithelium-mesenchyme boundary, drawn based on morphological differences between epithelial cells and mesenchyme cells in the pictures of the stained tissues, under enhanced contrast and/or brightness by using Photoshop, if needed. Dashed yellow boxes (d, f, n and p) indicate the higher magnification (i, j, s and t), respectively. TUNEL: green, and Hoechst: blue. T: typhlosole. At least 3 tadpoles were analyzed for each genotype. Bars: 100 µm. **B** Reduced or delayed cell death in *TRβ* knockout tadpoles. The TUNEL positive area in **A** was quantified and normalized against the Hoechst 33342 positive area. Different lower case letters denote statistically significant differences (*P* < 0.05) when compared in a single genotypes and asterisks (***) indicate a significant difference between the two genotypes (*P* < 0.001). *ns* no significant difference

When apoptosis was analyzed by using TUNEL labeling, we observed the peak level of apoptotic cells after 2 days of T3 treatment in wild type intestine (Fig. 7), as we reported before [68]. In contrast, in *TRβ* knockout tadpoles, the intestine had reduced number of apoptotic cells after 2 days of T3 treatment compared to the wild type animals (Fig. 7). Similar levels of apoptosis were observed between wild type and mutant intestine after 3 or 5 days of T3 treatment. Thus, *TRβ* knockout inhibited both adult epithelial stem cell development and/or proliferation and larval epithelial cell death during T3-induced intestinal remodeling, demonstrating a critical role of TRβ in intestinal metamorphosis.

## Conclusion

Amphibian metamorphosis mimics postembryonic development in mammals and offers a number of advantages for studying how T3 regulates vertebrate development. Earlier knockout studies, largely based on external morphological criteria, have revealed distinct effects of individual *TR* knockout in *Xenopus tropicalis. TRα* knockout does not affect embryogenesis but leads premature initiation of metamorphosis, i.e., reaching the onset of metamorphosis or stage 54 at younger age compared to wild type siblings, as determined based on hindlimb morphology [56, 57]. In addition, *TRα* knockout delays the metamorphic progression between stage 54 to stage 58, the early metamorphic climax, again as judged based on limb development. Thus, there is a critical role for TRα in limb development. However, *TRα* knockout has little effect on tail resorption. *TRβ* knockout also does not affect embryogenesis. It, on the other hand, has no effect on premetamorphic development or early metamorphosis, suggesting that it does not have a critical role in limb development (Fig. 1) [58, 59]. However, it causes severe delays in tail resorption, particularly notochord resorption [58, 59]. Interestingly, limited studies on the intestine, an internal organ, have revealed relative minor effects of *TRα* or *TRβ* knockout during natural metamorphosis, mainly a delay in intestinal remodeling relative to the external morphological changes in the knockout tadpoles [56–59]. This seems to contrast with the strong upregulation of *TRβ* during intestinal metamorphosis in both *Xenopus laevis* and *Xenopus tropicalis* [36, 54, 55], two related anuran species that undergo very similar intestinal remodeling [38, 47]. It is possible that the relatively minor effects observed for the *TR* knockouts during natural intestinal metamorphosis is in part due to comparing wild type and knockout animals of different ages (in order to match the stage) and/or due to distinct, developmental stage-dependent effects of the *TR* knockouts on the intestine and external organs that are used to determine the stages of the animals.

By using age- and stage-matched wild type and *TRβ* knockout tadpoles to study T3-induced metamorphosis, we have demonstrated here clearly that TRβ is critical for intestinal remodeling, from the reduction in intestinal length to the two major cellular changes, stem cell formation/proliferation and larval epithelial apoptosis via regulation of direct T3 target genes in the intestine. Our results have revealed the complications in studying gene function in vivo and in interpreting developmental outcomes due to gene-editing. It further highlights the advantage of the anuran metamorphosis model for investigating adult organ formation during postembryonic development.

## Materials and methods

### Experimental animals

Wild type adult *X. tropicalis* were purchased from Nasco or raised in the laboratory. Embryos and tadpoles were staged according to [69]. TRβ mutant frogs [*TRβ*^*(*+*/−)*^ and *TRβ*^*(−/−*)^] were reared in the 9 l plastic container until sexual maturation. All animal care and treatments were performed as approved by the Animal Use and Care Committee of Eunice Kennedy Shriver National Institute of Child Health and Human Development of the National Institutes of Health.

### Generation of *TRβ* knockout *Xenopus tropicalis* animals by using CRISPR-Cas9 genome editing technology and genotyping

CRISPR-short guide RNA (sgRNA) was designed as described [60, 70], to target exon 2 of *TRβ* gene, upstream of the DNA binding domain (Additional file 1: Table S1) (Fig. 1a). CRISPR-sgRNA injected embryos obtained from mating *TRα*(+ / −)TRβ(+ / +) and wild type frogs were reared to sexual maturity (F0-generation frogs). A sexually mature F0 frog was mated with a wild type frog, and their offspring were screened to identify tadpoles with wild type *TRα* by polymerase chain reaction (PCR) method [56] but mutant heterozygous *TRβ* by sequence analysis [*TRα(*+ / +)TRβ(+ / −)] (Fig. 1b). After *TRα*(+ / +)*TRβ(*+ / −) mutants were sexually mature (F1 frogs), female and male mutant frogs were primed with 20 U of human chorionic gonadotropin (Novarel) one day before egg laying. They were then boosted with another injection of 200 U of human chorionic gonadotropin on the second day for natural mating to obtain *TRβ* knockout [TRβ(− / −)] frogs (F2 generation). The resulting fertilized eggs/embryos were collected and reared for three days at 25 °C to reach the feeding stage (stage 45). The tadpoles were then transferred to a 4-L container and fed.

Tadpoles at indicated ages or stages were anesthetized with MS222 for photography, tail clipping, and body length measurement. For genotyping, tadpole tail tip (about 5 mm or less) was clipped and lysed in 20 µl QuickExtract DNA extraction solution (Epicentre) at 65 °C for 20 min. After incubating at 95 °C for 5 min, 1 µl of the DNA extraction solution was used for genotyping with PCR. A mutant line with a 5 base out-of-frame deletion in *TRβ*(Fig. 1b) was chosen for further studies. *TRβ* mutants were identified by PCR analysis of the genomic DNA with the forward primer F, 5′-TCAATGGAACCCTTTGGAGCTG-3′ and the reverse primer R, 5′-ACAGTTACAGGCATTTCCAGGC-3′ for 35 cycles of 94 °C for 10 s, 60 °C for 5 s, 72 °C for 45 s. The PCR products were analyzed by gel electrophoresis and purified by using QIAGEN PCR purification kit (Qiagen), followed by sequencing (Eurofins genomics).

### T3 treatment

Randomly selected stage 54 tadpoles were treated with or without 10 nM T3 for 18 h at 25 °C. The tadpole tail tip (about 5 mm or less) was cut for genotyping, and the rest of each animal was frozen in liquid nitrogen. The frozen tadpoles of the same genotype were combined together for RNA extraction. For the long term T3-treatment, wild type and *TRβ* knockout tadpoles at stage 54 were pooled into separate 4-l plastic containers and treated with 5 nM T3 for 0 (control), 2, 3, and 5 days at 25 °C. Half of the rearing water was replaced with fresh water with or without T3 every day. At least 3 tadpoles in each genotype were picked up and analyzed for each time point of T3 treatment.

### RNA extraction and qRT-PCR

The intestine from individual tadpoles was homogenized with RNeasy® Mini Kit 250 (Qiagen). The homogenates from at least three animals of each genotype were combined together for RNA extraction. The RNA concentration was measured by using a NanoDrop (Thermo Scientific). The same amount of RNA from each of the three genotypes (TRβ: wild-type, heterozygous and homozygous mutant) was reverse transcribed with the QuantiTect reverse transcription kit (Qiagen). The cDNA was analyzed by using the SYBR Green qPCR method. The PCR primers for indicated genes and internal control *rpl8* were described previously [56, 61] (note that *rpl8* mRNA expression were similar in all genotypes; data not shown) (Additional file 2: Table S2). All expression data were normalized against that of the internal control gene *rpl8*. The expression analyses were performed at least twice, with similar results.

### ChIP assay and quantitative PCR

Age-matched stage 54 tadpoles were randomly selected and treated with or without 10 nM T3 for 18 h at 25 °C in 4-l container as described above. They were then anesthetized in ice-water and genotyped by tail clipping. Five tadpoles of the same genotype were pooled together and homogenized for ChIP assay with anti-TR and anti-ID14 (negative control) antibodies as described previously [56, 71, 72]. The immunoprecipitated DNA was analyzed by using TaqMan quantitative PCR (Thermo Fisher Scientific) with gene specific primers/probes for the promoter and exon 5 of *TRβ*, as previously described [73].

### 5-Ethynyl-2′-deoxyuridine (EdU) labeling

EdU staining was performed as described [67]. Briefly, 1.25 and 10 μl of 10 mg/ml EdU were injected into stage 54 tadpoles. 30 min after injection, the tadpoles were sacrificed, and the intestine was isolated and fixed in 4% PFA/PBS for paraffin-sectioning. Tissue sections cut at 5 μm were subjected to EdU staining by using the Click-iT Plus EdU Alexa Fluor 594 Imaging kit (Thermo Fisher Scientific). EdU positive areas in epithelium were measured by using ImageJ software (National Institutes of Health).

### TUNEL assays

TUNEL (terminal deoxyribonucleotidyl transferase-mediated dUTP-biotin nick end labeling) assays were performed by using In Situ Cell Death Detection Kit (Roche) as described [74]. The fluorescent pictures for different colors and different sections were taken under the same settings and then analyzed by using ImageJ software at the same setting to measure the TUNEL positive cell area.

### Methyl green-pyronin Y (MGPY) staining

Tissue sections were stained with MPGY (Muto), a mixture of methyl green, which binds strongly to DNA, and pyronin Y, which binds strongly to RNA, for 5 min at room temperature according to supplier’s instructions. Adult epithelial stem/progenitor cells were intensely stained red because of their RNA-rich cytoplasm [51, 75, 76].

### Statistical analysis

Data are presented as mean ± SE. The significance of differences between groups was evaluated by one-way ANOVA followed by Bonferroni multiple comparison test or Student’s *t* test using Prism 5 (GraphPad Software).

## Supplementary information

**Additional file 1: Table S1.** . Primers used in *Xenopus tropicalis* thyroid hormone receptor β knockout experiments.

**Additional file 2: Table S2.** Primers used for qRT-PCR.

## Data Availability

Not applicable.
