# Supplementary material for: Thyroid hormone receptor beta is critical for intestinal remodeling during Xenopus tropicalis metamorphosis
Source: Cell Biosci. 2020 Mar 27;10:46. doi: 10.1186/s13578-020-00411-5 (PMC7099810; doi:10.1186/s13578-020-00411-5)
Supplement: Supplementary file 1 — Additional file 1: Table S1. . Primers used in Xenopus tropicalis thyroid hormone receptor β knockout experiments. [file 13578_2020_411_MOESM1_ESM.docx]

**Additional Table S1. Primers used in *Xenopus* *tropicalis* thyroid hormone receptor β knockout experiments.**

Name Primer sequence

CRISPR sgRNA 5′-TAATACGACTCACTATAGGGTACATACCCAGCT

ACTGTTTTAGAGCTAGAAATAGCAAG -3′

CRISPR-3´primer 5′-AAAAGCACCGACTCGGTGCCACTTTTTCAAGTTGATAA

CGGACTAGCCTTATTTTAACTTGCTATTTCTAGCTCTAAAAC-3′

Forward primer F 5′- TCAATGGAACCCTTTGGAGCTG -3′

Reverse primer R 5′- ACAGTTACAGGCATTTCCAGGC -3′
