# Supplementary material for: Thyroid hormone receptor beta is critical for intestinal remodeling during Xenopus tropicalis metamorphosis
Source: Cell Biosci. 2020 Mar 27;10:46. doi: 10.1186/s13578-020-00411-5 (PMC7099810; doi:10.1186/s13578-020-00411-5)
Supplement: Supplementary file 2 — Additional file 2: Table S2. Primers used for qRT-PCR. [file 13578_2020_411_MOESM2_ESM.docx]

**Additional Table S2. Primers used for qRT-PCR.**

Primer sequence

Gene Forward Reverse

*TRα* 5′- AAATGCATTGCCGTTGGCAT -3′ 5′- GCCGCTCTCGATTCTCTTCA -3′

*TRβ* 5′- CTCTAGCAGCTGATGGTGGG -3′ 5′- TGGGTTTTGTTCCTGGTGCT -3′

*klf9* 5′- GGCACAGGTGTCCTTATGCT -3′ 5′- AAGGGCGTTCACCTGTATGG -3′

*TH/bzip* 5′- CCAAGGGAAACGGGTGGCTT -3′ 5′- GTGCCACCTCTGCGGAAAGT -3′

*rpl8* 5′- AGAAGGTCATCTCATCTGCAAACAG -3′ 5′- CAATACGACCACCACCAGCAA -3′

*mmp2* 5′- TAGAGCACTCTCAAGATCCTGGAG -3′ 5′- AGGTCCAGGACGTGGTTTTTCCTT -3′

*mmp9th* 5′- CTTTGTTCGATGGGTCACTCTGCT -3′ 5′- ACGTTTCTGCATCCAGGTTG -3′

*mmp11* 5′- GGTTATGTGTGGCGCCTTCG -3′ 5′- AATGGGAAAGGGCCCAGAGG -3′

*mmp14* 5′- TGGAGATTTGCGGACACACACCTT -3′ 5′- ATTTGTCAGGGACTCCACATCGAG -3′

*casp3* 5′- AGCCCAAACTATTCTTCATCCA -3′ 5′- GATGAACCAGGAACCATTCATT -3′

*casp 9* 5′- GAGGAGTTTACGGAACAGATGG -3′ 5′- AGTCACCGAACATCCTTTGTCT -3′

*ror2* 5′- CCCACTGGGACAAATGGATA -3′ 5′- TCCTGACACTTTGCAGTGGA -3′

*wnt5a*  5′- CATCTCTCTCTGCTGCCTGA -3′ 5′- GCTGAGCCCCAATGATATAAA -3′
